# Editorial: Molecular innate immunity and AI data analysis in hepatic diseases

**DOI:** 10.3389/fimmu.2023.1276111

**Published:** 2023-08-24

**Authors:** Li Wang, Qian Liu, Min Yao

**Affiliations:** ^1^ Research Center for Intelligence Information Technology & Department of Immunology, Medical School, Nantong University, Nantong, China; ^2^ Nevada Institute of Personalized Medicine, University of Nevada, Las Vegas, NV, United States

**Keywords:** molecular innate immunity, machine learning, hepatic diseases, artificial intelligence, data analysis

Molecular innate immunity is the body’s initial immune mechanism responding to pathogen invasions, involving molecular signaling pathways and reactions to recognize and counter external threats. As a vital immune organ in the body, the liver contains diverse immune cells and molecular signaling pathways to safeguard against infections and diseases. Within the liver, molecular innate immunity plays a crucial role by identifying pathogen patterns, activating immune cells, defending against viruses, regulating immune inflammatory responses, and maintaining immune tolerance to protect the liver from infections and immune-related diseases (1). The application of Artificial Intelligence (AI) technology can be utilized to analyze liver molecular data, such as genomics and proteomics, to explore relevant genes and proteins associated with molecular innate immunity, along with pathways linked to immune responses. Additionally, AI techniques can assist in predicting molecular interactions within the liver, deciphering intricate immune signaling networks, and providing novel insights for the study and treatment of liver immune-related diseases (2). In this Research Topic, all accepted manuscripts have undergone rigorous peer review by researchers with a strong background in hepatology, oncology, and bioinformatics. These novel studies provide promising avenues for further exploration in liver cancer biology, drug screening, and precision medicine.

Among these accepted manuscripts, three of them utilized Gene Expression Omnibus (GEO) database for bioinformatics analysis. One study (Wang et al.) focused on biliary atresia (BA), a common cause of severe neonatal obstructive jaundice. The complex etiology of BA led to diverse pathological features and clinical outcomes. To address this, the study employed integrative clustering analysis using high-throughput datasets to classify BA into distinct molecular subtypes, aiming for personalized treatment strategies. By analyzing the GSE122340 RNA sequence dataset and collecting liver tissues, the study identified four molecular subtypes. These subtypes exhibited varying prognoses and distinct biological processes. One study (Bao et al.) suggested that tailored perioperative and preoperative treatments based on these subtypes could enhance BA prognosis. In research on the relationship between ischemic stroke (IS) and nonalcoholic fatty liver disease (NAFLD), they considered the potential cancer burden associated with NAFLD complicated by IS. Utilizing various datasets and methods such as weighted gene co-expression network analysis (WGCNA) and machine learning, the study identified immune-related candidate biomarkers for NAFLD with IS. A nomogram was developed to assess diagnostic efficacy and candidate biomarkers’ role in cancer, revealing six common immune-related genes shared by NAFLD and IS. Particularly, RRS1 was associated with prognosis, immune responses, and tumor characteristics, suggesting its potential for predicting cancer prognosis in NAFLD complicated by IS and aiding in diagnosis and treatment decisions. Another research (Bai et al.) addressed the insufficiently understood immune mechanisms underlying hepatic fibrosis (HF) and its associated inflammatory damage. Utilizing GSE84044 expression data, WGCNA identified crucial module genes and maps them to immune-related genes. Functional enrichment analyses reveal inflammation-related pathways enriched among the identified hepatic fibrosis immune genes (HFIGs). A protein-protein interaction network identified 10 hub genes, and immune infiltration analysis indicates correlations with immune cells. Most HFIG-associated pathways and hub gene expressions were validated, and potential therapeutic agents targeting the hub genes were explored through the CMAP platform. This research sheds light on immune mechanisms underlying HF and contributes to its diagnosis, prevention, and treatment in clinical settings.

Another interesting project (Li et al.) focused on tumor mutation burden (TMB), a predictive indicator for how cancer responds to immunotherapy. The relationships between TMB and various factors were explored across different cancers using multi-omics datasets from The Cancer Genome Atlas and datasets from cancer cohorts that underwent immune checkpoint blockade therapy. Several genes were identified with mutations associated with higher TMB and positive immunotherapy responses. Based on specific gene mutations, a TMB prognostic score was defined, demonstrating the predictive potential for cancer survival in immunotherapy settings. The study concluded that the molecular and clinical attributes linked to TMB have the potential to serve as valuable predictors for TMB and immunotherapy response, thereby holding promise for clinical applications in cancer management.

Among all manuscripts accepted, there is a review article (Chen et al.) about primary liver cancer (PLC). Systemic therapy is the main treatment for PLC, including surgical resection, immunotherapy, and targeted therapy. However, due to tumor heterogeneity, responses to the above drug therapies vary among individuals, indicating an urgent need for personalized treatment approaches for PLC. Organoids are 3D models derived from adult liver tissues or pluripotent stem cells. This review discussed the role of liver organoids in liver cancer research, particularly in reflecting the heterogeneity of liver cancer and restoring the tumor microenvironment (TME) by co-organizing tumor vasculature and stromal components *in vitro*. It also summarized recent advancements of liver organoids in liver cancer, covering generation methods, applications in precision medicine, and TME modeling. These advancements contributed to further exploration of liver cancer biology, drug screening, and precision medicine for PLC.

The future prospects for AI in hepatic molecular innate immunity are promising. AI technologies are poised to advance our understanding of intricate immune pathways and provide deeper insights into the molecular mechanisms underlying liver diseases. As AI algorithms continue to evolve, their capacity to analyze complex molecular data and model immune responses is expected to enhance our understanding of hepatic molecular innate immunity. Moreover, AI-fueled advancements could lead to the development of innovative immunotherapies targeting specific components of the liver’s molecular innate immune system, potentially revolutionizing the therapeutic landscape for immune-related liver disorders.

In conclusion, AI’s applications in hepatic molecular innate immunity encompass biomarker discovery, predictive modeling of immune responses, and a deeper comprehension of molecular pathways. As AI technologies progress, their integration into hepatic immunology research holds the potential to uncover new therapeutic strategies and drive forward our understanding of liver immune function.

## Author contributions

LW: Writing – original draft. QL: Formal analysis, Writing – original draft. MY: Writing – review & editing.
